# Microbial Community Changes across Time and Space in a Constructed Wetland

**DOI:** 10.1021/acsenvironau.4c00021

**Published:** 2024-07-26

**Authors:** Zeinah Elhaj Baddar, Raven Bier, Breann Spencer, Xiaoyu Xu

**Affiliations:** † Savannah River Ecology Lab, University of Georgia, PO Drawer E, Aiken, South Carolina 29802, United States; ‡ Warnell School of Forestry and Natural Resources, University of Georgia, University of Georgia, Athens, Georgia 30605, United States

**Keywords:** microbial community, constructed wetland, temporal variability, spatial variability, sulfur cycling

## Abstract

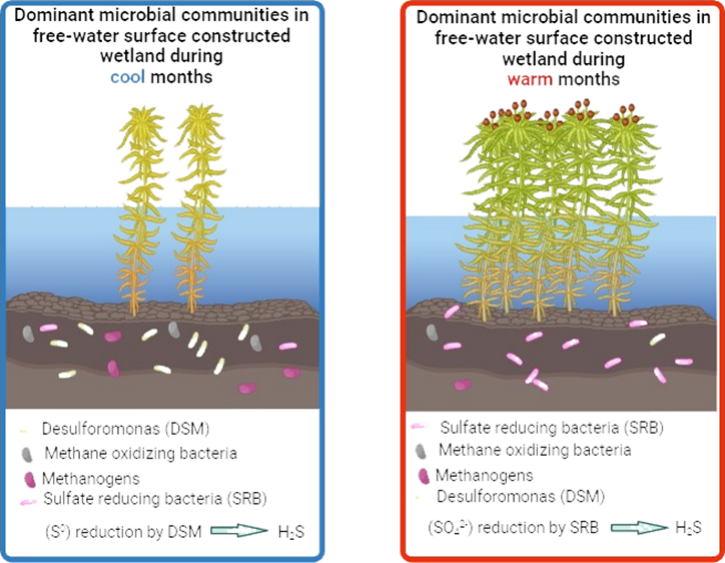

Constructed wetlands are artificial ecosystems designed to replicate natural wetland processes. Microbial communities play a pivotal role in cycling essential elements, particularly sulfur, which is crucial for trace metal fixation and remobilization in these ecosystems. By their response to their environment, microbial communities act as biological indicators of the wetland performance. To address knowledge gaps pertinent to the changes in trace metal bioavailability in relation to microbial activities in the H-02 constructed wetland, we performed this study to investigate temporal and spatial variations in microbial communities by using molecular biology tools. Quantitative polymerase chain reaction and next generation sequencing techniques were employed to analyze archaeal and bacterial groups associated with sulfur and methane cycling. Alpha diversity indices were used to assess species richness, evenness, and dominance. Results indicated high gene abundance of Desulfuromonas (5.37 × 10^6^ g.cell^–1^), methane oxidizing bacteria (6.92 × 10^6^ g.cell^–1^), and methanogenic microorganisms (3.02 × 10^5^ g.cell^–1^) during cool months. Warm months were marked by sulfate reducing bacteria dominance (3.31 × 10^6^ g.cell^–1^), potentially due to competitive interactions and environmental conditions, higher temperatures, and lower redox potential. Spatial variability among microbial groups was insignificant, but trends in gene abundance indicated complex factors influencing these groups. Next generation sequencing data demonstrated Firmicutes as the most abundant phylum with over 50% regardless of the season or sampling location. Cool months exhibited higher alpha diversity than warm months. Overall, this study showed that seasonal changes significantly impacted the microbial communities in the H-02 constructed wetland that are associated with the sulfur cycle and eventually trace metal biogeochemistry, revealing two distinct mechanisms of the sulfur cycle between the two main seasons, whereas spatial variability effects were not conclusive.

## Introduction

Constructed wetlands are artificial ecosystems designed to replicate the ecological processes of natural wetlands.^1−3^ Microbial communities play a pivotal role as significant catalysts for the cycling of essential elements and metals within these engineered systems,^4^ especially in free water surface constructed wetlands. The sulfur cycle is a major ecological process in which a consortium of microorganisms orchestrates the reduction and oxidation of sulfur compounds in the environment. One major aspect of the sulfur cycle is its role in the fixation/remobilization of trace metals in the environment.^5^ In free water surface constructed wetlands, the anaerobic reduction of sulfur compounds is highly desirable as this would result in the formation of insoluble metal sulfides, thus helping the wetland system act as a sink for these contaminants.^6,7^ Likewise, several archaeal and bacterial groups are involved in methane (CH_4_) production and consumption in the wetland system, which directly impacts global warming.^8^

Microbial communities within constructed wetlands shift according to changes in environmental conditions, such as temperature, availability of nutrients, carbon sources, redox potential, and pH.^9^ These fluctuations in microbial communities could serve as a biological indicator for the performance of the wetland.^10^ Therefore, it is crucial to monitor and interpret these changes to provide a comprehensive understanding of biogeochemical cycling within these ecosystems.

The changes in microbial communities could manifest in both time and space. Spatial changes could indicate the presence of gradients, patches, and microniches within the system,^11^ leading to spatial heterogeneity in both the horizontal and vertical dimensions. In the work of Semenov et al.,^12^ it was found that the diversity in the microbial communities heavily relied on changes in the physiochemical properties and the vegetation of a free water surface wetland. Similarly, shifts in physiochemical characteristics, such as changes in organic matter content and nitrogen levels, led to alterations in bacterial communities within a horizontal flow constructed wetland.^13^ Temporal variabilities could indicate the diversity and adaptability of the microbial communities in response to seasonal shifts, which could have been driven by drastic changes in temperature and recurring cycles of wetting and drying,^14,15^ or changes in contaminant levels.^16^

In this study, several molecular biology tools were used to study temporal and spatial changes in microbial communities in the H-02 wetland system, which is a free water surface constructed wetland. To this end, we investigated the abundance of specific archaeal and bacterial groups, especially those associated with sulfur and CH_4_ using a quantitative polymerase chain reaction (qPCR) tool, which helped us quantify specific genetic sequences within microbial DNA. Likewise, the high-throughput technology of next generation sequencing (NGS) was implemented to investigate the taxonomical changes in microbial communities in the H-02 wetland system over time and space. Species diversity indices such as Shannon, Simpson’s, and Chao1 provided information on species richness, evenness, and dominance within the H-02 wetland system.

Based on our previous work at the H-02 wetland system,^17^ we suggested the oxidation of sulfur to be more dominant during cool months, while sulfate reduction was the major mechanism taking place during the warm months. Therefore, in this work, we hypothesized that microbial communities associated with sulfur oxidation and the subsequent reduction of these compounds will be more dominant during the cold months, whereas sulfate reducing bacteria (SRB) will be more dominant during the warm months. We also hypothesized significant shifts in microbial community structures with the sampling location.

## Methods

### Sampling Site

In 2007, the H-02 constructed wetland was established on the Savannah River Site (SRS) in Aiken, South Carolina, with the primary purpose of serving the Tritium facility. This wetland operates as a free water surface system, responsible for treating both the wastewater generated by Tritium facility processes and managing stormwater runoff. The wastewater initially exits the facility through two primary source pipes, entering a retention basin where it remains for 3 days. This duration represents the hydraulic retention time (HRT),^18^ as illustrated in the accompanying figure (Figure S1). Subsequently, the water flows from the retention basin to the influent pool, and from there, it is directed to the splitter box. At the splitter box, the wastewater is evenly divided between two adjacent wetland cells known as WC1 and WC2. Each of these wetland cells is rectangular, measuring 22.4 m in width and 100 m in length.

The wastewater enters the wetland cells through the inflow site and exits from the outflow end. The treated effluent from the wetland cells ultimately discharges into Upper Three Runs Creek, eventually reaching Savannah River (Figure S1).

### Sample Collection, Storage, and Processing

This study constituted a comprehensive 10-month investigation spanning both the cooler period (from October 2021 to February 2022) and the warmer season (from May 2022 to September 2022), as extensively detailed in ref (19). In brief, passive water samplers were deployed once a month throughout the specified time frame mentioned above, with each deployment lasting 8 days. During each sampling event, *in situ* measurements of pH and temperature were taken using a hand-held pH meter Oakton pH 5+ (Oakton, Eutech Instruments, Vernon Hills, IL), and measurements of reduction/oxidation (RO) potential were taken using an Oakton ORPTestr 10 Waterproof Pocket ORP Tester (Oakton, Eutech Instruments, Vernon Hills, IL). Each month, four samples were collected, with one from each wetland cell and location as follows: one sample from the inflow location in WC1, one from the outflow location in WC1, one from the inflow location in WC2, and one from the outflow location in WC2. In total, 40 samples were collected throughout the duration of the study.

On the day of passive sampler retrieval, sediment samples were collected using 100 mL capacity sterile Whirl-Pak bags, covering a sampling depth of 15 cm below the flocculant–water interface to encompass both the organic-rich flocculent layer and soft and clayey mineral sediments. Sample collection was performed by scooping floc and sediment into the bags manually to create one composite sample. Careful precautions were taken to prevent excessive oxygen exposure within the sample bags. This involved expelling excess air from the bags and sealing them while they were submerged beneath the water surface. Minimizing the headspace and thus reducing oxygen exposure were crucial to preserve the microbial community as closely as possible to its natural state. To maintain the structural integrity of the microbial community, the samples were promptly transferred to the laboratory while they were on ice. Upon returning to the laboratory, the samples were immediately placed in a freezer at −80 °C to prevent microbial activities.

Following the collection process, samples were processed in preparation for microbial community analysis. This involved freeze-drying the samples (Labconco Corporation; Kansas City, MO, USA) until constant weight was achieved, sieving them through a 2.0 mm sieve, followed by thorough homogenization. Subsequently, the samples were shipped on ice, ensuring their overnight delivery to Microbial Insights, Inc. (Knoxville, TN, USA) for qPCR and NGS analyses.

### Quantitative Polymerase Chain Reaction (qPCR)

Quantitative polymerase chain reaction (qPCR) was performed on a QuantStudio 12K Flex Real-Time PCR System (Applied Biosystems, Grand Island, NY). All qPCR experiments included appropriate negative (no DNA) and positive control reactions. No amplification was detected in negative controls (the Ct value was greater than the total number of reaction cycles). Quantitative PCR results were reported per each wetland cell (WC1 and WC2) and per sampling site (inflow (IF) and outflow (OF)) during the cold months and warm months.

### Next Generation Sequencing (NGS)

DNA extractions were performed, and 16S rRNA gene amplicon sequencing was conducted following amplification using universal bacterial primers that were designed for the high-throughput MiSeq platform (Illumina) and target the 16S rRNA V3 and V4 regions.^20^ Operational taxonomic unit (OTU) assignment and analyses of community alpha and beta diversity were analyzed according to the Quantitative Insights Into Microbial Ecology (QIIME2) bioinformatics pipeline.^21^ Taxonomic classification of 16S rRNA gene amplicon reads was performed with the Illumina 16S Metagenomics application, which utilizes the RefSeq RDP 16S v3 database and a high-performance implementation of the Ribosomal Database Project (RDP) Classifier algorithm.^22^

### Statistical Analysis

Principal Component Analysis (PCA) was conducted using R-Studio version 4.2.2^23^ to analyze pH, RO, temperature, and qPCR data. We also utilized Python 3.11.6, made available by the Python Software Foundation (https://www.python.org/), to concatenate the NGS data. The sequencing depth of the samples was evaluated using a rarefaction curve, and samples that did not meet the specified minimum threshold of 20,000 sequences were considered to have insufficient sequencing depth and, as a result, were excluded from further analysis unless mentioned otherwise (Figure S2).

The analysis of significant patterns among the qPCR data was performed using the nonparametric Freidman test, which is equivalent to the parametric repeated measures ANOVA, and Nemenyi test was used to perform posthoc pairwise comparisons. Comparison of qPCR data between the two seasons (warm versus cool) and the two locations (inflow versus outflow) were performed using *t* tests. All these tests were performed using R-Studio, 4.2.2 at a significance cutoff *p*-value of 0.05.

Rarefaction indices were calculated using R-Studio 4.2.2. The major Phyla for each season were selected based on those that were most consistently dominant. The selection process involved arranging the phyla based on their abundance and selecting the top eight in the cold and warm seasons. One-way ANOVA was used to compare the common major phyla between the seasons (cool and warm) and the locations (inflow and outflow).

For figure generation, OriginLab,^24^ R-Studio 4.2.2, and Microsoft Excel were implemented. The α diversity indices: Shannon, Simpson’s, and Chao1 were calculated and performed by Microbial Insights Inc. on the genera present in the full data set of NGS data.

## Results and Discussion

### Temporal and Spatial Variability in Functional Guilds and Taxa in Wetland Sediments

**Figure 1:**
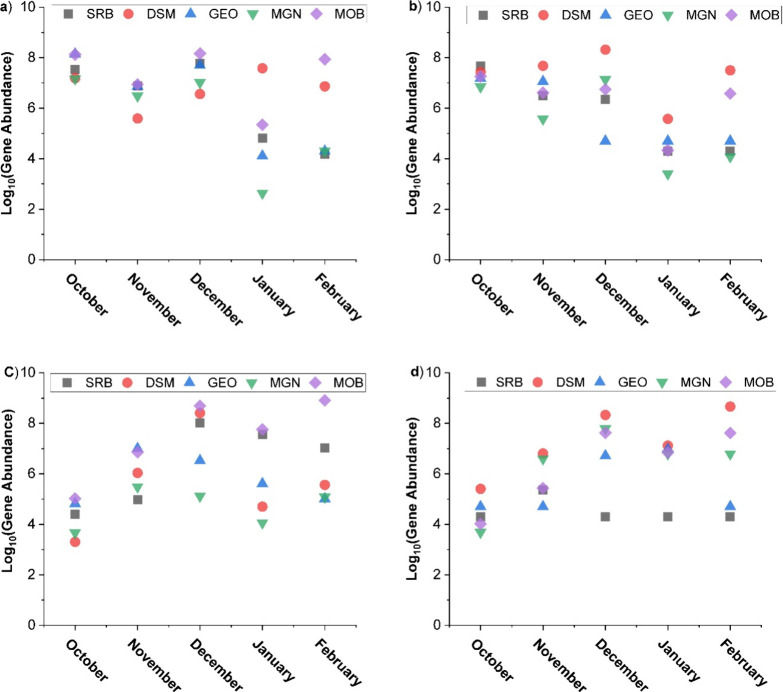
Gene abundances from qPCR data during the cool season for (a) WC1 at the inflow (IF), (b) WC1 at the outflow (OF) sites, (c) WC2 at the inflow (IF), and (d) WC2 at the outflow (OF) sites. Sulfate reducing bacteria (SRB), Desulfuromonas (DSM), Geobacter (GEO), methanogens (MGN), and methane oxidizing bacteria (MOB).

The overall analysis of the general trend in the data was significant; however, most post hoc pairwise comparisons were not (Table S1). During the cool months and at the inflow location, both wetland cells WC1 and WC2 showed a high gene abundance of methane oxidizing bacteria (MOB) almost every month of the season (Figure 1a,c). Whereas at the outflow site, Desulfuromonas (DSM) had noticeably high abundance throughout the cool season (Figure 1b,d).

In the cool months, methane oxidizers had almost the same trend as methanogens (Figure 1, Table S1). The synergistic nature of that dynamic could be attributed to fact that methanotrophs use CH_4_, which is a byproduct of methane producers, as their main source of energy.^25^ For example, as reported by Conrad and Rothfuss, 1991,^26^ 80% of CH_4_ produced in rice fields by MGN was found to be consumed by MOB. The coexistence of MGN and MOB was also reported by Franchini et al., 2015,^11^ even though MGN are considered strictly anaerobes^27^ while MOB are aerobic organisms. The authors inferred that the presence of microniches likely explained this phenomenon. The coexistence of both microorganisms in our study also suggests the presence of microniches that could occur in the sediment pore water of the H-02 wetland and requires further investigation.

Desulfuromonas gene abundance was noticeably higher at the outflow site for both wetland cells and exceeded the abundance of the other microorganisms (Figure 1b,d, Table S1). Sulfur respiration was discovered after the isolation of Desulfuromonas in the work of Pfenning and Bieble, 1976.^28^ In the present study, the increased abundance of Desulfuromonas at the outflow site for both cells implies a higher rate of sulfur oxidation, whereas the prevalence of Geobacter (GEO) appeared to peak during the earlier cool months and gradually declined as the season progressed.

**Figure 2:**
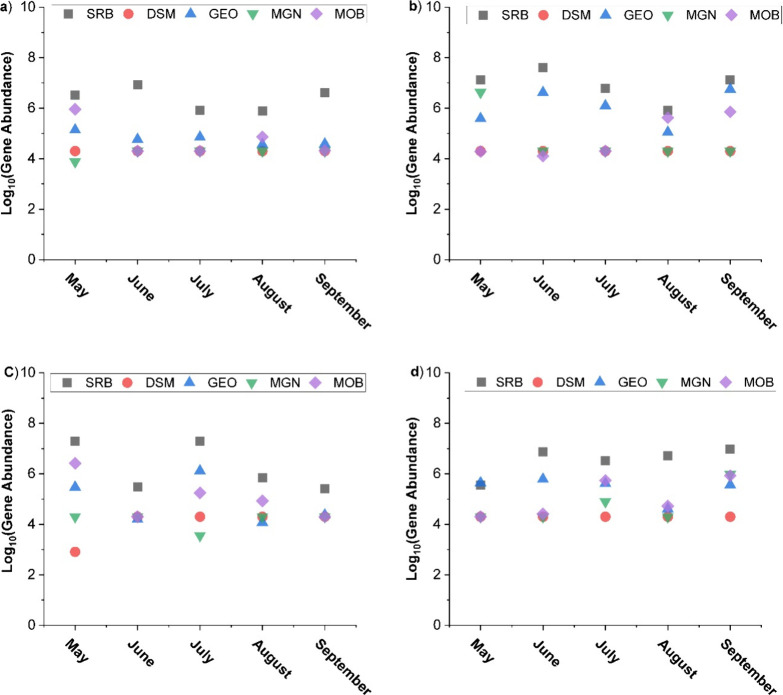
Gene abundances from qPCR data during the warm season for (a) WC1 at the inflow (IF), (b) WC1 at the outflow (OF) sites, (c) WC2 at the inflow (IF), and (d) WC2 at the outflow (OF) sites. Sulfate reducing bacteria (SRB), Desulfuromonas (DSM), Geobacter (GEO), Methanogens (MGN), and methane oxidizing bacteria (MOB).

During the warm months, SRB were consistently dominant across different locations and cells (Figure 2a–d, Table S1), while MOB and MGN populations showed a general decrease. This finding initially contradicts some studies that suggest an increase in CH_4_ production by MGN populations with rising temperatures.^29^

The observed SRB dominance can be attributed to competitive interactions with the MOB and MGN for organic substrates. Our previous work,^30^ which explored microbial community changes in H-02 wetland sediments over time, similarly indicated a shift toward greater SRB abundance at the expense of MGN and MOB abundance, aligning with the current findings. Likewise, He et al., 2015,^25^ reported that the presence of SRB suppressed CH_4_ production. It is important to note that this competition did not fully eliminate MGN, possibly due to the abundance of organic matter during the warm months. In fact, the findings of Sela-Adler et al., 2017,^31^ reported a coexistence between SRB and MGN rather than outright elimination. However, the decline in MGN population observed in our study during the warm months may involve more intricate interactions, including competition for acetate between MGN and iron-reducing microorganisms such as Geobacter, which have been reported in several studies.^32−34^

### Temporal and Spatial Variations in Wetland Microbial Populations

Compared to the cool months, which exhibited a population of 5.37 × 10^06^ ± 25 g.cell^–1^, the Desulfuromonas population was significantly (*p* <0.0001) lower during the warm months (1.70 × 10^04^ ± 2 g.cell^–1^) because sulfur oxidation is less likely as O_2_ solubility decreases with increasing temperatures, implying less sulfur oxidation and more sulfur staying in reduced forms (Table S2). Qin et al., 2022,^35^ and Elhaj Baddar et al., 2023,^36^ recently reported that sulfate concentrations in the pore water of the H-02 constructed wetlands were significantly lower during the warm months. So, it is highly likely that the higher sulfate concentrations in the cool months were not due to increased SO_4_^2–^ production, but rather to the lower rates of SO_4_^2–^ removal by the smaller SRB population. Being strictly anaerobes, the SRB population would shrink in cool months due to the increase in dissolved O_2_ content in the pore water. In the present study, the population of SRB was significantly lower in the cool months (5.50 × 10^05^ ± 30 g.cell^–1^) compared to the warm months (3.31 × 10^06^ ± 5 g.cell^–1^) (*p* = 0.041) (Table S2). Our findings agree with the work of Kusel et al., 2008,^37^ which reported a similar pattern of increased SO_4_^2–^ especially at temperatures as low as 4 °C, and they attributed that to the lower rate of sulfate consumption by the smaller SRB populations. In addition, Desulfuromonas, especially the acetate oxidizing species, seem to thrive at lower temperatures.^38^ All data are presented as mean gene abundance ± standard deviation.

Likewise, MGN and MOB both showed significantly (*p* = 0.0126 and <0.0001, respectively) lower gene abundance during the warmer season (2.95 × 10^04^ ± 5 and 8.13 × 10^04^ ± 5 g.cell^–1^, respectively) versus (3.02 × 10^05^ ± 35 and 6.92 × 10^06^ ± 25 g.cell^–1^, respectively) during the cool season. Whereas the Geobacter population did not significantly change between the two seasons (6.46 × 10^05^ ± 20 g.cell^–1^) in the cool season versus (1.86 × 10^05^ ± 6 g.cell^–1^) during the warm season (Table S2).

Spatial variability was not significant among the different microbial groups when comparing the qPCR data between the inflow and outflow locations (Table S3). Despite this finding, the general trend in gene abundance was still significant, which suggests a more complex dynamic of several factors affecting the abundance of the selected microbial groups.

### Temporal and Spatial Variations in Physiochemical Properties

The changes in pH, RO, and temperature were monitored throughout the study (Figures S3–S5). Significant changes in pH values were observed between the inflow and outflow locations for both wetland cells (WC1:8.98 ± 0.90 versus 6.57 ± 0.21, *p* = 0.0002; WC2:8.53 ± 1.04 versus 6.41 ± 0.11, *p* = 0.0002). However, there were no statistically significant changes in the pH values over time. The consistently higher pH values for both wetland cells at the inflow site are expected since the water entering the wetland from the retention basin is alkaline^17^ (Figure S3). On the other hand, the pH values at the outflow site for both cells were consistently slightly acidic to neutral regardless of the season (Figure S3). Likewise, no statistically significant temporal changes were observed for RO values; however, spatial variability was statistically significant, and at both wetland cells, when comparing the inflow and the outflow sites as following (WC1:72.3 ± 28.7 mV versus 107.4 ± 28.6 mV, *p* = 0.0312; WC2:73.4 ± 28.1 mV versus 104.8 ± 30.5 mV, *p* = 0.0140) (Figure S4). All data are presented as the mean ± standard deviation.

### Effects of Physiochemical Properties on Functional Guilds and Taxa

**Figure 3:**
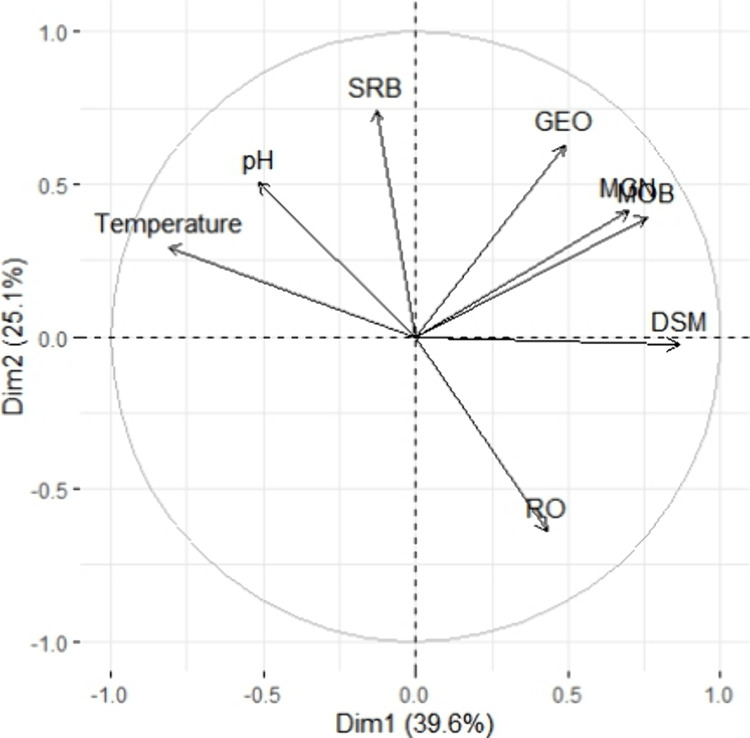
Principal components analysis (PCA) of qPCR data (sulfate reducing bacteria (SRB), Desulfuromonas (DSM), Geobacter (GEO), Methanogens (MGN), methane oxidizing bacteria (MOB), and pH, temperature, and redox potential (RO).

Principal Component Analysis (PCA) was applied to qPCR data, including the numerical variables: pH, temperature, and RO. The analysis yielded two principal axes capturing 64.7% of the data’s variability (Figure 3, Table S4). Notably, the strongest significant and positive correlation between temperature and the microbial groups was with SRB (*r* = 0.36, *p* <0.0001, Figure 3, Table S4), indicating that SRB thrived in warmer environments.

The analysis further revealed a negative correlation between RO levels and SRB populations (*r* = −0.2, *p* = 0.0002, Table S4, Figure 3). This underscores the sensitivity of these communities to increasing RO values, which are indicative of prevailing aerobic conditions during the cool season. A strong significant negative correlation was found between MOB population and temperature (−0.60, *p* <0.0001), whereas a weak yet significant positive correlation was observed between RO and MOB (*r* = 0.01, *p* <0.001, Table S4, Figure 3). The only significant correlation of pH was that with the MGN population (*r* = −0.22 *p* = 0.003), indicating a sensitivity of this population to alkaline pH. No significant correlations were observed between pH, RO, or temperature and Geobacter (Table S4, Figure 3).

**Figure 4:**
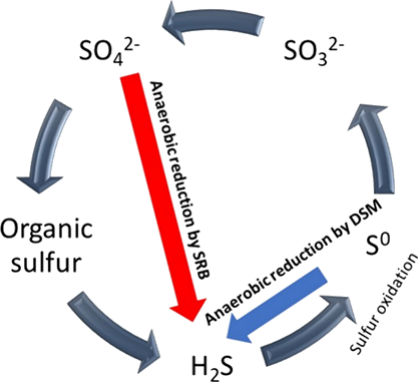
Proposed mechanisms of sulfur reduction in the H-02 wetland system. Red and blue arrows refer to the proposed dominant mechanism during the warm and cool months, respectively. Sulfate reducing bacteria are denoted as (SRB) and Desulfuromonas as (DSM).

The unexpected shifts in sulfur reduction from Desulfuromonas during the cool months to SRB during the warm months shed light on an important aspect of the sulfur cycle in the H-02 constructed wetland system. Despite the lower activity of SRB during cool months and the risk of heavy metal release due to the oxidation of H_2_S, through the activities of Desulfuromonas, anaerobic reduction of elemental sulfur helps keep adequate levels of H_2_S in the H-02 wetland system to keep bioavailable levels of trace metals as low as possible (Figure 4). This finding corroborates our previous work^17^ that suggested the dominance of sulfur oxidation during the cool months, whereas the warm months favored the anaerobic reduction of sulfate.

### Microbial Community Structure of Wetland Sediment

**Figure 5:**
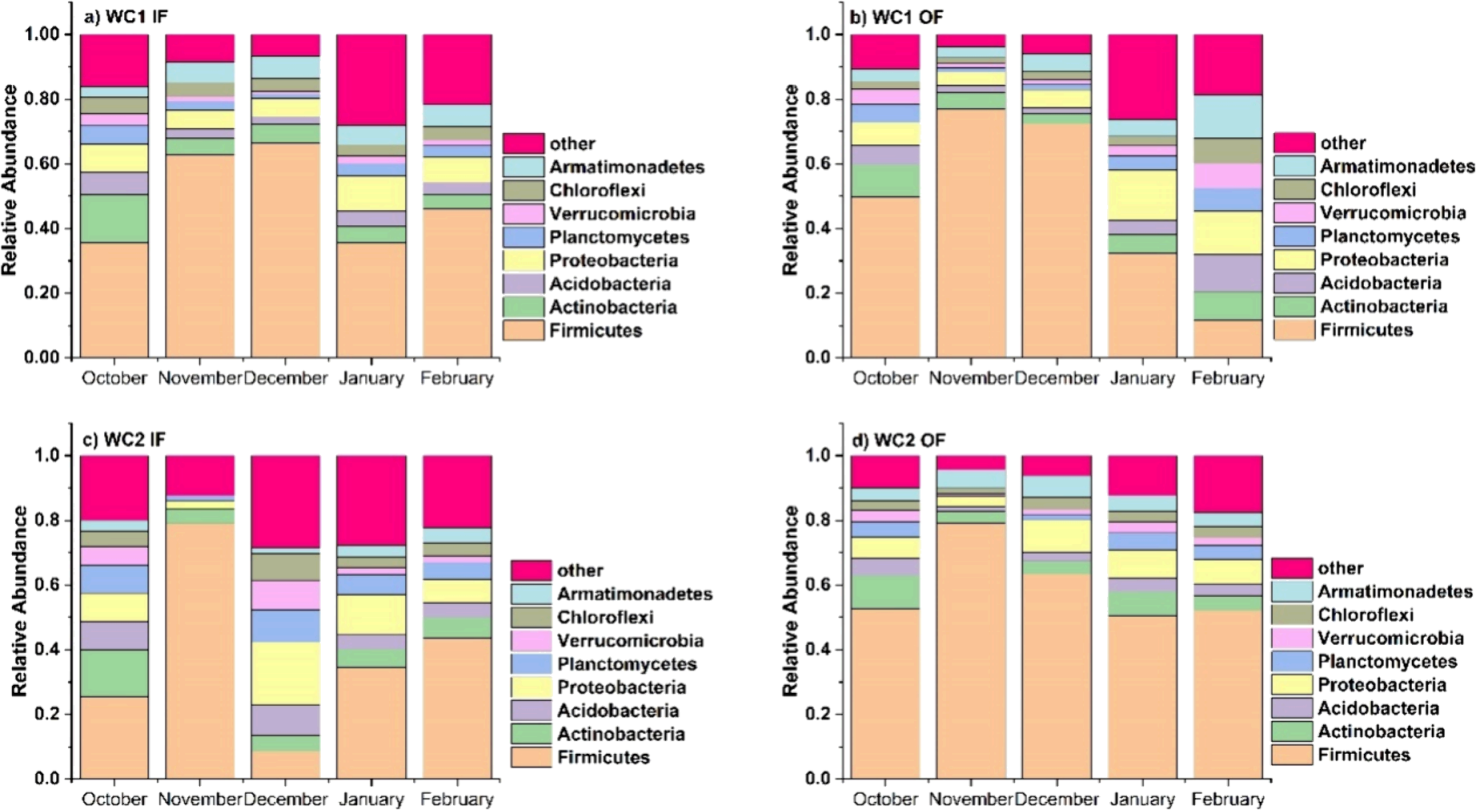
Changes in the relative abundance of the most dominant phyla in the sediments of the H-02 wetland during the cool months are at (a) WC1 at the IF location, (b) WC1 at the OF location, (c) WC2 at the IF location, and (d) WC2 at the OF location.

During the cool months and for both wetland cell locations, Firmicutes were the dominant phylum, with a relative abundance of ≥50% compared with the rest of the phyla of interest (Figure 5). The highest relative abundance of Firmicutes occurred in WC2 at the outflow site in November and was 79.2% (Figure 5d). The exceptions were during October for both WC1 and 2 at the inflow location (Figure 5a,c), December for WC2 at the inflow location (Figure 5c), January for both WC1 and 2 at the inflow location (Figure 5a,c), and February at WC1 at the outflow location (Figure 5b).

Proteobacteria relative abundance was at its highest (19.5%) during December at WC2 at the inflow location (Figure 5c). The relative abundance of Actinobacteria was more noticeable at the beginning of the cool season, especially during October for almost all cells and locations (Figure 5a–d). Verrucomicrobia presence was more pronounced at WC1 at the outflow location during February (Figure 5b), and at WC2 at the inflow location during December (Figure 5c). In December at WC2 at the inflow site (Figure 5c), Planctomycetes, Verrucomicrobia, Chloroflexi, and Armatimonadetes exhibited a similar relative abundance, each accounting for approximately 10%. This uniform distribution of these taxa is indicative of the elevated diversity observed in this sample.

**Figure 6:**
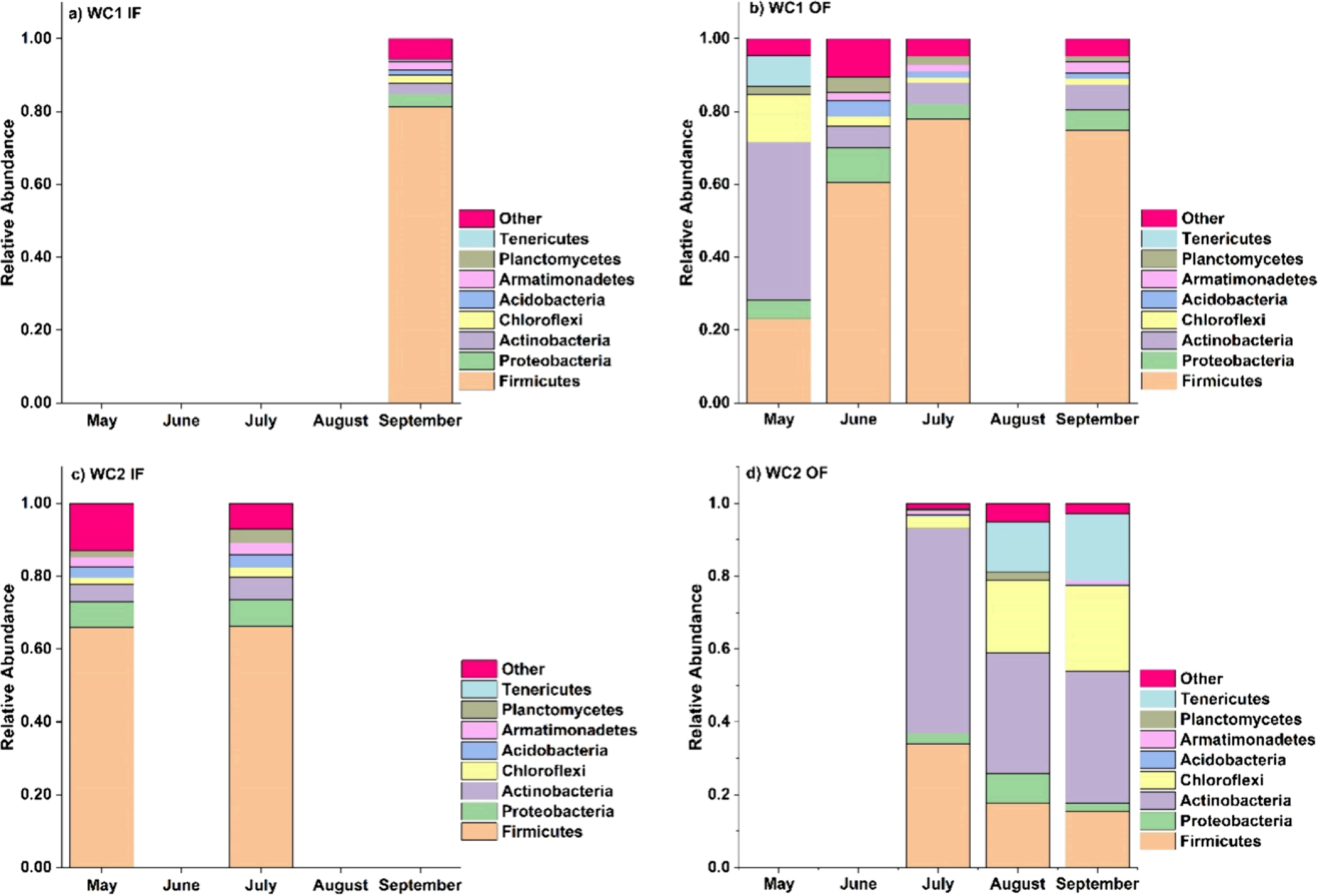
Changes in the relative abundance of the most dominant phyla in the sediments of the H-02 wetland during the warm months at (a) WC1 at the IF location, (b) WC1 at the OF location, (c) WC2 at the IF location, and (d) WC2 at the OF location.

In the warm months, phylum Firmicutes was relatively more abundant at 3 out of 4 sites: WC1 at the inflow (Figure 6a), WC1 at the outflow (Figure 6b), and WC2 at the inflow location (Figure 6c). The highest relative abundance of Firmicutes was 81.3% at WC1 at the inflow site during September (Figure 6a). Interestingly, Actinobacteria phylum seemed to coincidently be more relatively dominant whenever Firmicutes was less dominant (Figure 6b,d), which took place only at the outflow location for both cells. In fact, Actinobacteria had the highest relative abundance (56.2%) at WC2 at the outflow location during July (Figure 6d). The peak relative abundance of Chloroflexi and Proteobacteria occurred in September and June, respectively, reaching 23.6 and 9.6%, at WC2 at the outflow site (Figure 6d) and WC1 at the outflow site (Figure 6b).

**Figure 7:**
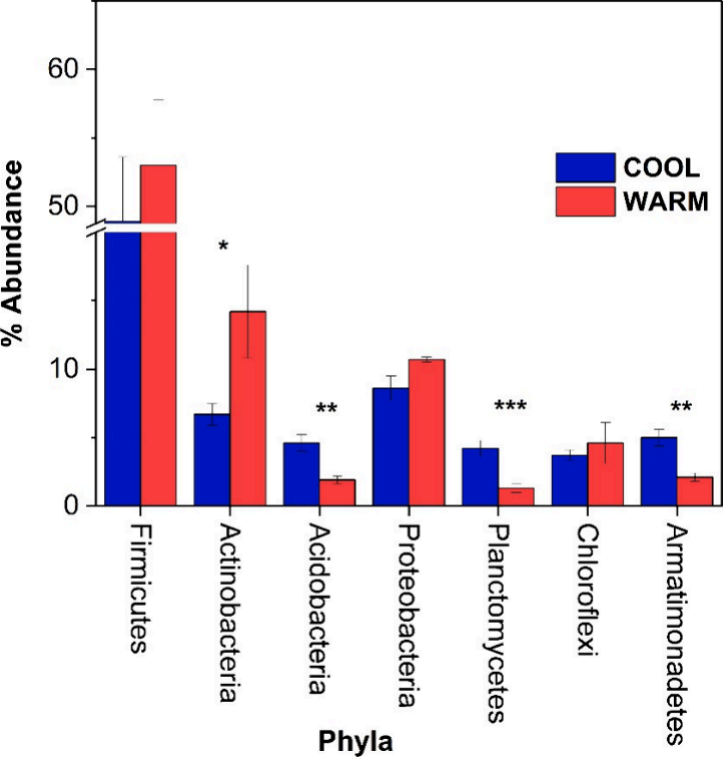
Relative abundance of common phyla in the H-02 constructed wetland during the cold and warm seasons. Alpha = 0.05. (*, **, and *** refer to *p* values <0.05, 0.001, and 0.0001, respectively).

We tested potential effects of seasonal and spatial variations on the most common phyla from all of the samples. Of all the seasonal and spatial variation comparisons of microbial relative abundances, the only statistically significant comparison observed was between the common phyla during the two seasons: cool and warm (Figure 7). More specifically, the statistically significant differences were observed for Actinobacteria (6.7% ± 0.8 cool months versus 14.2% ± 3.4 warm months, *p* = 0.04), Acidobacteria (4.6% ± 0.6 cool months versus 1.9% ± 0.3 warm months, *p* <0.001), Planctomycetes (4.2% ± 0.6 cool months versus 1.3% ± 0.3 warm months, *p* <0.0001), and Armatimonadetes (5.0% ± 0.6 cool months versus 2.1% ± 0.3 warm months, *p* <0.001), data represented as mean ± standard error of the mean, *p*-value at α = 0.05 (Figure 6). Overall, cool months had significantly higher abundance of Acidobacteria, Planctomycetes, and Armatimonadetes compared to the warmer months, which showed significantly higher abundance of Actinobacteria. These results require further studying of which specific microorganism, at the genus level, had a higher representation in each phylum.

Overall, four of the major phyla detected in the H-02 wetland (Firmicutes, Proteobacteria, Acidobacteria, and Actinobacteria) were also detected in several wetlands globally, as shown in the meta-analysis study conducted by Lv et al.,^39^ based on available 16S rRNA gene sequences recovered from several previous studies found in the GenBank database.

### Diversity Indices

There was an agreement between patterns of diversity based on Shannon and Simpson indices for both the cool months (Table 1) and the warm months (Table 2). The lowest Shannon index and richness values in species were for WC2 at both sites, inflow and outflow, during November, where at the outflow site, Shannon and Simpson indices were, respectively, 3.75 and 0.91, whereas at the inflow site, although excluded due to not meeting the sequence depth criteria, the Shannon index was 3.93 and the Simpson index was 0.96 (Table 1). Likewise, both samples scored the lowest and third lowest Chao1 index values, respectively, 555 and 226 for WC2 at the outflow and inflow sites. The highest Shannon and Simpson index values were, respectively, 5.19 and 0.98 for WC2 at the inflow site and coincided with the highest Chao1 index value of 1600 (Table 1). These results agree with the richness and evenness of taxonomic phyla within these samples reported by the 16S rRNA data (Figure 5c,d).

**Table 1:** Genus Diversity Indices for Samples Collected during the Cool Months^a^

**sample**	**Shannon**	**Simpson**	**Chao1 predicted genera**
WC1 IF October	4.74	0.97	1164
WC1 OF October	4.53	0.96	1320
WC2 IF October	4.80	0.98	1423
WC2 OF October	4.41	0.95	1182
WC1 IF November	4.32	0.95	988
WC1 OF November	4.05	0.93	987
WC2 IF November*	3.93	0.96	226
WC2 OF November	3.75	0.91	555
WC1 IF December	4.39	0.97	853
WC1 OF December	4.27	0.95	1025
WC2 IF December	5.19	0.98	1600
WC2 OF December	4.27	0.95	941
WC1 IF January	4.47	0.95	1142
WC1 OF January	4.65	0.96	1341
WC2 IF January	4.59	0.95	1306
WC2 OF January	4.49	0.95	1150
WC1 IF February	4.41	0.96	1091
WC1 OF February*	4.43	0.97	408
WC2 IF February	4.39	0.95	1084
WC2 OF February	4.34	0.95	1001

a WC1 and WC2: wetland cells 1 and 2. IF: inflow location, OF: outflow location. Samples marked with * did not meet the sequence depth threshold of 20,000 sequences.

**Table 2:** Genus Diversity Indices for Samples Collected during the Warm Months^a^

**sample**	**Shannon**	**Simpson**	**Chao1 predicted genera**
WC1 IF May*	4.80	0.98	804
WC1 OF May	3.32	0.90	1665
WC2 IF May	4.32	0.94	1568
WC2 OF May*	4.72	0.96	918
WC1 IF June*	4.31	0.96	847
WC1 OF June	4.69	0.95	1531
WC2 IF June*	3.83	0.95	265
WC2 OF June*	4.58	0.96	769
WC1 IF July*	3.78	0.91	683
WC1 OF July	3.90	0.90	1218
WC2 IF July	4.16	0.92	1165
WC2 OF July	2.83	0.75	742
WC1 IF August*	3.71	0.95	242
WC1 OF August*	4.31	0.97	382
WC2 IF August*	4.19	0.96	310
WC2 OF August	3.38	0.90	1665
WC1 IF September	4.08	0.95	979
WC1 OF September	3.99	0.92	1796
WC2 IF September*	4.49	0.97	456
WC2 OF September	2.84	0.85	1597

a WC1 and WC2: wetland cells 1 and 2. IF: inflow location, OF: outflow location. Samples marked with * did not meet the sequence depth threshold of 20,000 sequences.

In general, wetland sediment contained lower diversity during the warmer months than during the cooler months based on Shannon and Simpson indices (4.42 ± 0.30 cool months versus 4.01 ± 0.58 warm months for Shannon index; 0.96 ± 0.02 cool months versus 0.93 ± 0.05 for Simpson index) (Table S5). The lowest Shannon and Simpson index values, 2.83 and 0.75, respectively, were recorded in July at the WC2 outflow location. The highest diversity, although the sample is excluded, was noted in May at the WC1 inflow site, with Shannon and Simpson indices of 4.8 and 0.98, respectively (Table 2). These findings are consistent with the prevalence of either Firmicutes or Actinobacteria in the sampled sites during the warmer months (Figure 6), possibly outcompeting less dominant groups and, thereby, lowering diversity. It is important to note that patterns of Shannon and Simpson diversity index values differed when compared to the Chao1 index values. For instance, despite having lower Chao1 index values of 742.05 and 1596.78 for the samples with the lowest Shannon and Simpson diversity, which were observed in WC2 at the outflow site in July and WC2 at the outflow site in September, respectively, the Chao1 value for the most diverse sample, recorded in WC1 at the inflow site in May, was relatively low at 804.28 (Table 2). This disparity may be attributed to the Chao1 index’s sensitivity to rare species, potentially leading to an underestimation of diversity within the sample. For example, the sample with the highest Shannon and Simpson diversity (WC1 inflow, May) had a relatively low Chao1 index (804), while samples with lower Shannon and Simpson values (WC2 outflow, July and September) showed higher Chao1 values (742 and 15977 respectively) (Table 2). This discrepancy may be due to the Chao1 index's greater sensitivity to rare species, which could lead to an underestimation of diversity in the WC1 inflow sample.

Ma et al., 2020,^40^ showed that, in soil samples, the warmer months significantly increased the α diversity indices compared to the cooler months, possibly due to environmental variables such as pH, temperature, and trace metal content. Likewise, it was found that pollution and pH negatively affected the diversity of microbial communities in acid mine drainage contaminated natural wetlands.^41^ On the other hand, some studies indicated no significant seasonal effects on the diversity indices, but reported notable changes in the community structure in wetlands^42^ and soils.^43^ The diversity in microbial communities is tied to an array of environmental and ecological conditions that could complicate the interpretation of the results and perhaps mask the effect of seasonality on the microbial community structure in any system.

Spatial variability by the sampling location (IF versus OF) was not observed for Shannon and Chao1 indices, whereas Simpson index data showed slightly higher diversity (0.96 ± 0.018) at the IF sampling location when compared to that at the OF location (0.93 ± 0.052) (Table S5).

## Conclusions

This study was performed to evaluate the shifts in microbial communities in response to spatial and temporal changes in the H-02 constructed wetland system. Our previous work suggested that sulfur oxidation could have been more active during the cool months.^17^ The data from the present study supported that hypothesis whereby the abundance of Desulfuromonas was notably higher during the cool months compared to the warmer ones. In addition, SRB were more dominant during the warm months, most likely due to their positive association with the temperature in the environment, as shown in the PCA results. The implication of this finding is closely tied to the proper functioning of the H-02 wetland. The increase in sulfur production due to the increased oxidation of H_2_S during the cool months was counteracted by the anaerobic reduction of sulfur by Desulfuromonas, keeping H_2_S levels high enough to sustain trace metal sequestration and subsequent precipitation. Anaerobic reduction of SO_4_^2–^ was the dominant mechanism by which the H-02 wetland sustained adequately enough levels of H_2_S. While there were significant temporal changes in the microbial populations at the H-02 wetland, the results with regard to spatial changes were not as fully conclusive. Diversity indices corroborated the NGS data in highlighting the months when the microbial communities showed either high or low diversity, and hence require further investigation of the role of environmental conditions and trace metal concentrations and forms on specific genera.

The coexistence of anaerobic MGN with aerobic MOB in this study suggests the presence of microniches in the H-02 wetland. Future research should focus on elucidating the dynamics of microbial activities at the micron scale. Further investigation is required to analyze and identify the specific microorganisms contributing to key processes in the sulfur cycle and trace metal biogeochemical cycling, particularly at the genus level. Additionally, future studies should aim to elucidate the temporal dynamics of the microbial communities observed in this study with greater precision. To achieve this, more intensive sampling efforts will be necessary to capture the evolving trends in microbial communities over time, thereby enhancing the reliability and comprehensiveness of future research endeavors.

The prevalence of Desulfuromonas, in particular, has important energy-related applications. Microbial cells are gaining more attention as green sources of energy, and Desulfuromonas is among the few genera that showed very promising capabilities in generating electricity through the oxidation of sulfide at the anode side of microbial fuel cells.^44^

## Data Availability

Raw data files are available from Xiaoyu Xu at: xiaoyuxu@uga.edu, upon reasonable request.

## Supporting Information Available

Figures of the H-02 wetland system layout, refraction index analysis, changes in pH, changes in redox potential (RO), and changes in temperatures with month in the H-02 wetland system; tables of statistical analyses results for qPCR data, PCA, and diversity indices (PDF)
