# Supplementary material for: Microbial Community Changes across Time and Space in a Constructed Wetland
Source: ACS Environ Au. 2024 Jul 26;4(6):307–16. doi: 10.1021/acsenvironau.4c00021 (PMC11583098; doi:10.1021/acsenvironau.4c00021)
Supplement: Supplementary file 1 — vg4c00021_si_001.pdf [file vg4c00021_si_001.pdf]

Microbial Community Changes across time and space in a constructed wetland

Zeinah Elhaj Baddar [1*], Raven Bier [1], Breann Spencer [1], and Xiaoyu Xu [1,2]

1 Savannah River Ecology Lab, University of Georgia, PO Drawer E, Aiken, SC 29802, USA

2Warnell School of Forestry and Natural Resources, University of Georgia, University of Georgia, Athens, GA, 30605, USA

* Corresponding author: Zeinah Elhaj Baddar (Zeinah.Baddar@uga.edu)

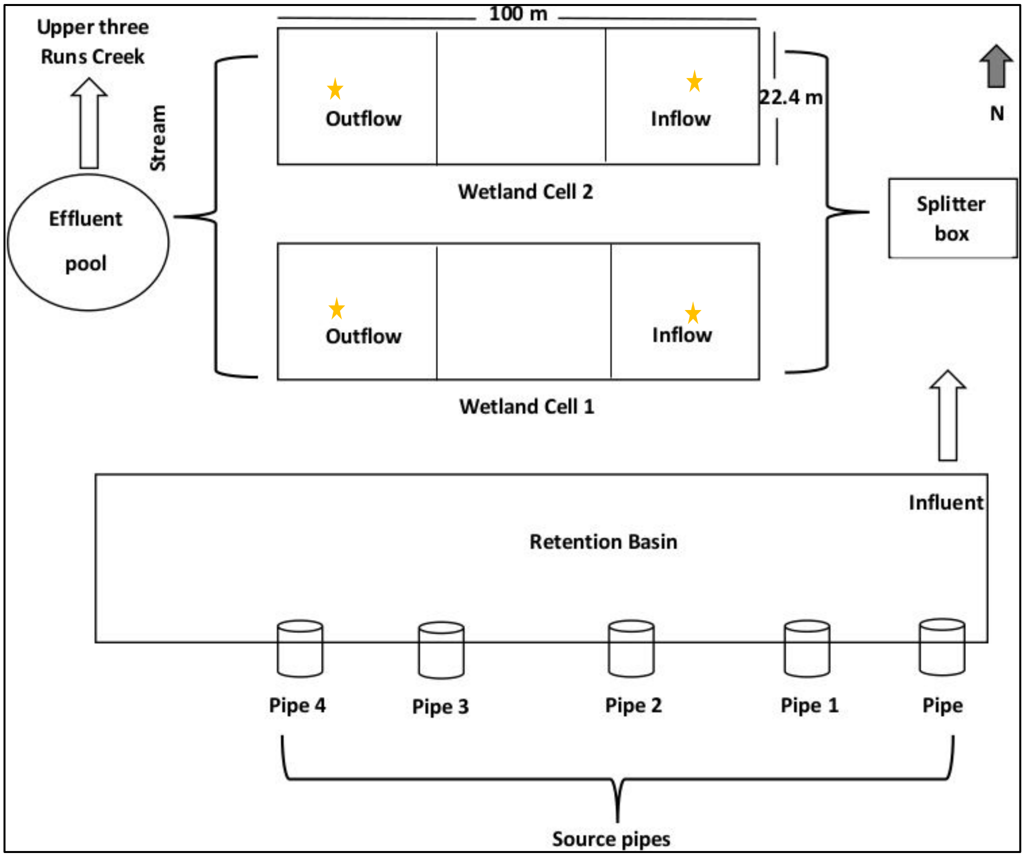

Fig. S1 The H-02 wetland system, the stars represent the sampling locations. Adopted from Xu and Mills. 2018[1], and Baddar et al. 2021 [2]. Reproduced or adapted with permission from [3]. © 2023 The Authors. *Environmental Toxicology and Chemistry* published by Wiley Periodicals LLC on behalf of SETAC.

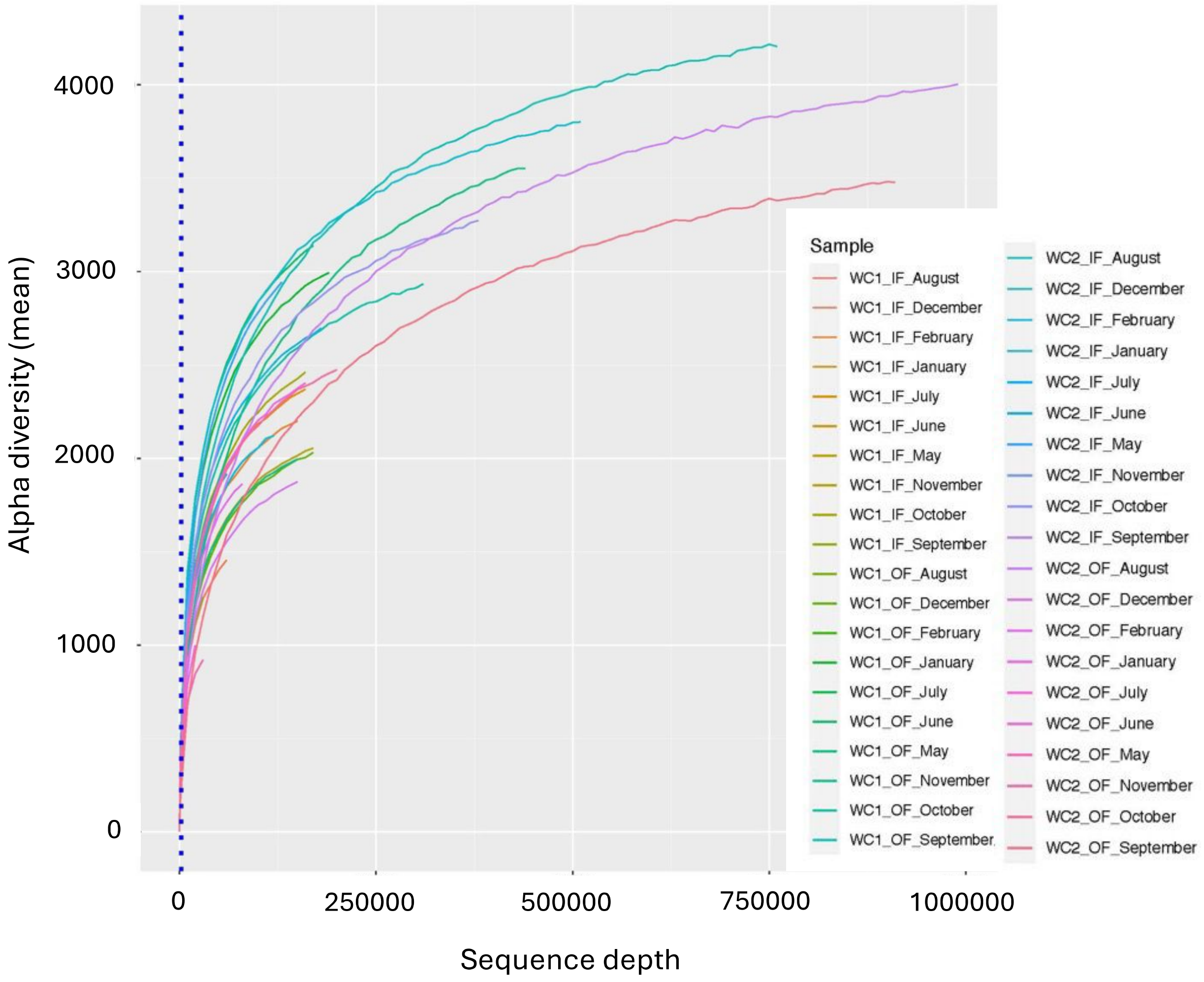

Fig. S2 Refraction index analysis for samples collected from the H-02 wetland system, wetland cell (WC1, WC2), and location (inflow, IF, and outflow, OF).

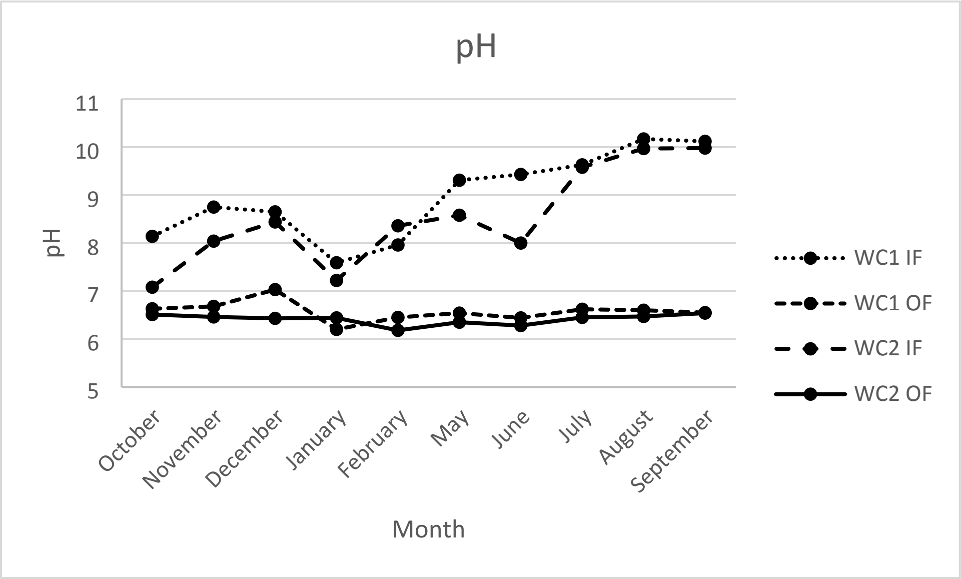

Fig. S3 Changes in pH with month, wetland cell (WC1, WC2), and location (inflow, IF, and outflow, OF)

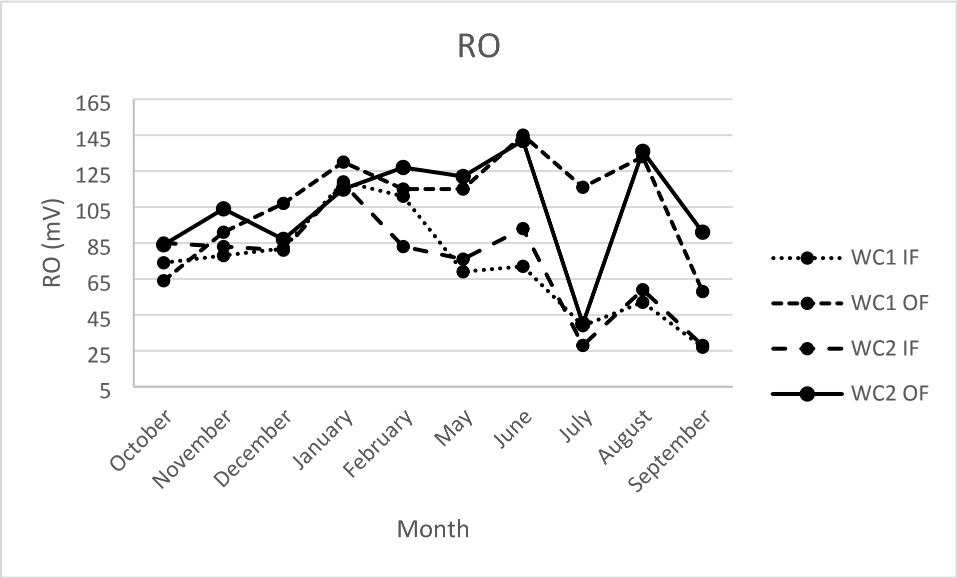

Fig. S4 Changes in redox potential (RO) with month, wetland cell (WC1, WC2), and location (inflow, IF, and outflow, OF)

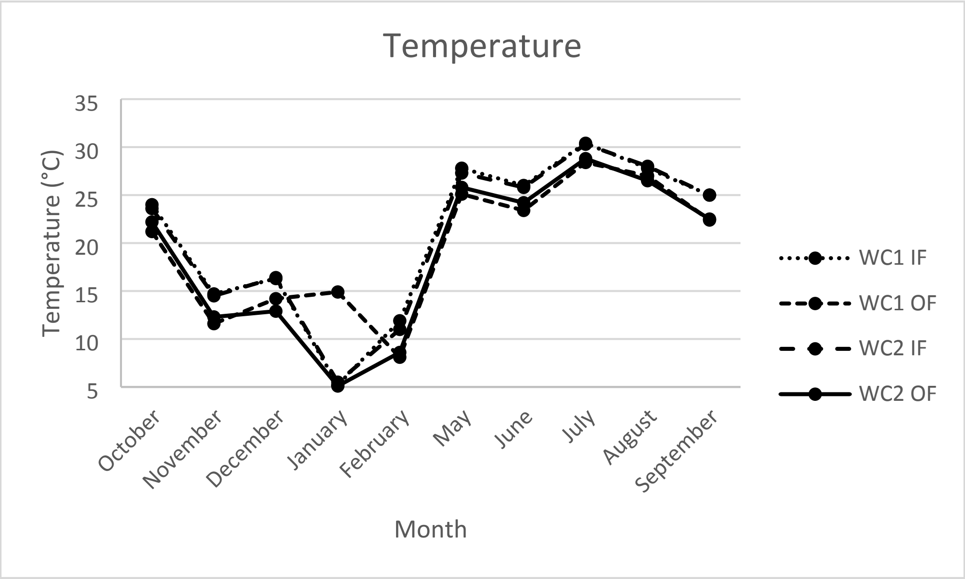

Fig. S5 Changes in temperatures with month, wetland cell (WC1, WC2), and location (inflow, IF, and outflow, OF)

Table S1 Freidman test results performed on qPCR data. IF: inflow location, OF: outflow location, WC1: wetland cell 1, WC2: wetland cell 2. Sulfate reducing bacteria (SRB), Desulfuromonas (DSM), Geobacter (GEO), methanogens (MGN), methane oxidizing bacteria (MOB). NA=not available, NS: not significant

Cool Months				Warm Months			
WC1		WC2		WC1		WC2	
IF	OF	IF	OF	IF	OF	IF	OF
Overall *p*-value = 0.0629	Overall *p*-value = 0.0280	Overall *p*-value = 0.0299	Overall *p*-value = 0.0151	Overall *p*-value = 0.00173	Overall *p*-value = 0.00610	Overall *p*-value = 0.0123	Overall *p*-value = 0.00690
Post-hoc *p*-value = NA	Post-hoc *p*-value = 0.031 (MGN and SRB)	Post-hoc *p*-value = 0.0061 (GEO and SRB)	Post-hoc *p*-value = NS	Post-hoc *p*-value = NS	Post-hoc *p*-value = NS	Post-hoc *p*-value = NS	Post-hoc *p*-value = NS

Table S2 Results of t-test performed on qPCR data based on season (warm and cool). Data represented as mean gene abundance ± standard deviation (g.cell^{-1}). Sulfate reducing bacteria (SRB), Desulfuromonas (DSM), Geobacter (GEO), methanogens (MGN), methane oxidizing bacteria (MOB).

	Cool	Warm	*p*-value
SRB	5.50E+05 ± 30	3.31E+06 ± 5	0.041
DSM	5.37E+06 ± 25	1.70E+04 ± 2	< 0.0001
GEO	6.46E+05 ± 20	1.86E+05 ± 6	0.121
MGN	3.02E+05 ± 35	2.95E+04 ± 5	0.0126
MOB	6.92E+06 ± 25	8.13E+04 ± 5	< 0.0001

Table S3 Results of t-test performed on qPCR data based on location (inflow and outflow). Data represented as mean gene abundance (g.cell^{-1}) ± standard deviation. Sulfate reducing bacteria (SRB), Desulfuromonas (DSM), Geobacter (GEO), methanogens (MGN), methane oxidizing bacteria (MOB).

	Inflow	Outflow	*p*-value
SRB	6.32 ± 1.16	5.94 ± 1.24	0.33
DSM	5.17 ± 1.48	5.79 ± 1.71	0.23
GEO	5.41 ± 1.24	5.67 ± 0.93	0.45
MGN	4.64 ± 1.14	5.31 ± 1.36	0.10
MOB	6.13 ± 1.65	5.62 ± 1.25	0.27

Table S4 Correlation matrix for numerical variables in the PCA, *, **, *** refer to *p*-values <0.05, < 0.001, and < 0.0001, respectively.

	**SRB**	**DSM**	**GEO**	**MGN**	**MOB**	**pH**	**RO**	**Temperature**
**SRB**	1.00	-0.25	0.46	0.13*	0.14***	0.22	-0.20*	0.36***
**DSM**	-0.25	1.00	0.26***	0.56	0.64	-0.27	0.22	-0.74
**GEO**	0.46	0.26***	1.00	0.55	0.46	-0.18	-0.04	-0.10
**MGN**	0.13*	0.56	0.55	1.00	0.54	-0.22*	0.03	-0.30
**MOB**	0.14***	0.64	0.46	0.54	1.00	-0.04	0.01**	-0.60***
**pH**	0.22	-0.27	-0.18	-0.22*	-0.04	1.00	-0.72**	0.34
**RO**	-0.20*	0.22	-0.04	0.03	0.01**	-0.72**	1.00	-0.41
**Temperature**	0.36***	-0.74	-0.10	-0.30	-0.60***	0.34*	-0.41**	1.00

Table S5 Pairwise comparison of diversity indexes between the seasons (cool VS. warm) and location (Inflow (IF) and outflow (OF)) using t-test of unequal variances at α = 0.05. Data presented as mean ± standard deviation.

Comparison / Index	Shannon	Simpson	Chao 1
**Season** (Cool)	4.42 ± 0.30	0.96 ± 0.02	1039 ± 324
**Season** (Warm)	4.01 ± 0.58	0.93 ± 0.05	980 ± 518
p-value	0.009	0.036	0.66
**Location** (IF)	4.35 ± 0.37	0.96 ± 0.02	910 ± 421
**Location** (OF)	4.09 ± 0.53	0.93 ± 0.05	1110 ± 417
p-value	0.10	0.030	0.14

References

1Xu, X. Y., & Mills, G. L. (2018). Do constructed wetlands remove metals or increase metal bioavailability? *Journal of Environmental Management*, *218*, 245-255. https://doi.org/10.1016/j.jenvman.2018.04.014

2Baddar, Z. E., Peck, E., & Xu, X. Y. . . (2021). Temporal deposition of copper and zinc in the sediments of metal removal constructed wetlands. *Plos One 16(8)*. https://doi.org/doi:10.1371/journal.pone.0255527

3Baddar, Z. E., Xu, X. Y., & Spencer, B. (2023). Spatiotemporal Changes in Trace Metal Bioavailability in the Sediment Pore water of a Constructed Wetland Using Passive Pore water Samplers. *Environmental Toxicology and Chemistry*, *42*(12), 2726-2736. https://doi.org/10.1002/etc.5745
